# Coronavirus Disease 2019 (COVID-19) in Neonates and Children From China: A Review

**DOI:** 10.3389/fped.2020.00287

**Published:** 2020-05-15

**Authors:** Yuanqiang Yu, Pingyang Chen

**Affiliations:** Department of Pediatrics, The Second Xiangya Hospital, Central South University, Changsha, China

**Keywords:** neonates, children, coronavirus, COVID-19, SARS-CoV-2

## Abstract

At the end of 2019, a novel coronavirus began to spread in Wuhan, Hubei Province, China. The confirmed cases increased nationwide rapidly, in part due to the increased population mobility during the Chinese Lunar New Year festival. The World Health Organization (WHO) subsequently named the novel coronavirus pneumonia Coronavirus Disease 2019 (COVID-19) and named the virus Severe Acute Respiratory Syndrome Coronavirus-2 (SARS-CoV-2). Soon, transmission from person to person was confirmed and the virus spread to many other countries. To date, many cases have been reported in the pediatric age group, most of which were from China. The management and treatment strategies have also been improved, which we believe would be helpful to pediatric series in other countries as well. However, the characteristics of neonatal and childhood infection still have not been evaluated in detail. This review summarizes the current understanding of SARS-CoV-2 infection in neonates and children from January 24 to May 1, as an experience from China.

## Introduction

From 2002 to 2003, the outbreak of severe acute respiratory syndrome (SARS) in Guangzhou, China, caused a global epidemic, which brought widespread concern about a coronavirus epidemic (1). Later, another zoonotic coronavirus pathogen, known as the Middle East Respiratory Syndrome Coronavirus (MERS-CoV), spread in the Middle East from 2012, and the disease was named Middle East respiratory syndrome (MERS) (2). A new type of coronavirus was recently reported in Wuhan, Hubei Province, China, which also causes severe respiratory disease. The outbreak of the disease began in China, and has brought a heavy burden on the whole world (3). Considering that newborns and children are susceptible to infectious diseases, the prevalence of the disease among them is the subject of much attention. The strategy in dealing with the cases in neonates and children, as well as a healthy pediatric age group, form elaborate plans in fighting against the novel coronavirus disease. Such experience from the Chinese government and hospitals may also benefit the rest of the world. Here, we review the advances of current research from January 24 to May 1 in the epidemiology, clinical manifestations, management, and treatment of this disease in newborns and children. Cases and recommendations in the pediatric age group from China, published either in English or Chinese, are included.

## Search Strategy

References for this review were identified through searches of PubMed for articles published from January 1, 2003, to May 1, 2020, by use of the terms “coronavirus,” “neonate,” “children,” “COVID19,” and “SARS-CoV-2.” Relevant articles published between 2003 and 2020 were identified through searches in the authors' personal files. We further searched the recent online articles from the COVID-19 Academic Research Communication Platform of Chinese Medical Journal Network, where the latest relevant Chinese articles are published. Some news and policies from WHO are also involved for the latest information of COVID-19. Articles published in English and Chinese were included. Articles resulting from these searches and relevant references cited in those articles were reviewed.

## Epidemiology of COVID-19

### Background

In late December 2019, Wuhan, Hubei Province, China reported for the first time a large cluster of patients with unexplained pneumonia associated with the wholesale Huanan Seafood Market (4). Subsequently, the Chinese Center for Disease Control and Prevention (China CDC) sent a rapid response team to identify the source of the pneumonia virus cluster, and then isolated and sequenced a new coronavirus, named 2019 novel coronavirus (2019-nCoV) (5). The World Health Organization (WHO) subsequently named the novel coronavirus pneumonia Coronavirus Disease 2019 (COVID-19) and named the virus Severe Acute Respiratory Syndrome Coronavirus-2 (SARS-CoV-2). Since the virus was transmitted to additional family members by a family (including a 10-year-old asymptomatic child) returning to Shenzhen from Wuhan, widespread transmission had quickly emerged from person to person (6). As the Chinese Lunar New Year festival approached, population mobility had increased and the virus had spread rapidly throughout the country (7). Although the incubation period values are very similar to SARS or MERS, the transmission of COVID-19 may be more rapid, because of the possibility of transmission during the incubation period (8, 9). Specifically, some patients may be completely asymptomatic carriers who have passed symptom-based screening, but RT-PCR was positive for SARS-CoV-2 (10, 11). WHO then identified the incident as a public health emergency of international concern (PHEIC) on January 30, and on March 11 assessed that COVID-19 could be characterized as a pandemic (12, 13).

### Epidemiology in Pediatric Group

Among the previously diagnosed family from Shenzhen, the 10-year-old asymptomatic boy was the first child confirmed infected with the virus (6) Later, on January 19, 2020, a 7-year-old boy with a fever and cough was reported in Shanghai after visiting his grandfather in Wuhan (14). The symptoms of COVID-19 appear to be less severe in infants and children than in adult patients, similar to the SARS-CoV infection (15–17). The first case series report in children showed that the interval between symptom onset and exposure to index symptomatic case ranged from two to 10 days (mean 6.5 days), which suggests a longer incubation period for SARS-CoV-2 infection in children (18). Furthermore, the mean number of secondary symptomatic cases in a household exposure setting was 2.43, similar to the basic reproductive number in earlier research on adults (18, 19). Most cases in children were likely to expose themselves to family members or other children with COVID-19, and linked directly or indirectly to Hubei Province, indicating that extra protection of children in families is urgently needed, especially those linked to Wuhan (16, 20). A 13-month-old child was reported as the first severe case on February 8 (21). Furthermore, a 17 day-old newborn was reported as the first neonatal infection on February 5, testing positive with SARS-CoV-2 in pharyngeal swabs and anal swabs (22). In another case, pharyngeal swab testing was positive 36 h after birth (23). China CDC reported that, as of February 8, 2020, of 2,135 pediatric patients <18-years old, 728 (34.1%) were laboratory-confirmed with COVID-19 and 1,407 were (65.9%) suspected (16). Nearly 1% of the total population of patients reported were children under 10-years old (24). Two deaths were reported in children. One was a 14-year-old boy and the other was a 10-month-old child (16, 25). Seven neonates were reported with a positive nucleic acid test, and three with elevated IgM antibodies to SARS-CoV-2 and negative nucleic acid tests (22, 23, 26–30). Therein, no death but one severe case was involved (26). Therefore, we call for preventive and protective measures for pregnant women, newborns, and children against the spread of the disease as soon as possible.

### Global Response

The rapid and close collaboration between epidemiologists, virologists, biologists, clinicians, and drug researchers during the COVID-19 outbreak is commendable. Early in the disease outbreak, different models estimated the basic reproduction number R0 of SARS-CoV-2, calling for public health interventions and preparation plans (31–34). The Chinese government had taken emergency measures, such as organizing medical teams to support Wuhan, controlling population movements, establishing more hospitals for the treatment of COVID-19, and developing specific vaccines (35). A nationwide school closure had also been ordered, and children were confined in their homes with online courses offered (36). Based on the epidemiological data, different countries have adopted different measures to limit the spread of the novel coronavirus as well, such as issuing travel warnings, interrupting flights, prohibiting nationals from going to severely affected countries, and adopting 14 day quarantine rules for nationals from affected areas (37, 38).

## Virology and Pathogenesis

### Virology

Coronaviruses (CoVs) are pathogens that can infect humans, domestic animals, and much wildlife, and can invade multiple organ systems such as the respiratory, gastrointestinal, liver, and central nervous systems. This subfamily includes four genera: *alpha-coronavirus, beta-coronavirus, gamma-coronavirus*, and *delta-coronavirus* (39). SARS-CoV-2 is the seventh CoV known to infect humans and cause respiratory diseases. It belongs to the clade 2 of the subgenus sarbecovirus, Orthocoronavirinae subfamily of *beta-coronavirus*, and is different from SARS-CoV and MERS-CoV (5, 40).

The novel coronavirus was first isolated from human airway epithelial cells and observed under a transmission electron microscope (5). Electron micrographs showed the distinctive spikes(S) (about 9–12 nm) and corona of the virus particles. In ultrathin sections of the human airway epithelium, virus particles were filled in membrane-bound vesicles in the cytoplasm or distributed in the extracellular matrix (5). Researchers had found that the genome had 89% nucleotide homology with bat SARS-like CoVZXC21, and even 96.2% sequence identity with BatCoV RaTG13 (41, 42). Another study also suggests that pangolins may be possible hosts of SARS-CoV-2 (43).

In addition, the SARS-CoV-2 genomic sequence is far from SARS-CoV (about 79%) and MERS-CoV (about 50%) (40, 41). The amino acids in different proteins have also been replaced accordingly, which further explains the structural and functional differences between SARS-CoV-2 and SARS-CoV (44). However, SARS-CoV-2 has a similar receptor-binding domain structure to SARS-CoV, which is located in the S1 conserved domain and critical for determining host tropism and transmission capabilities (40). They may use the same cell-targeted receptor angiotensin-converting enzyme 2 (ACE2), and Cryo-EM showed that SARS-CoV-2 S had 10- to 20-fold higher affinity to bind with ACE2 than SARS-CoV S (41, 45, 46). Further research and understanding of the structure of SARS-CoV-2 would better facilitate the development of vaccines as well.

### Pathogenesis

It has to be mentioned that the specimens from the respiratory and gastrointestinal tracts were detected as SARS-CoV-2, which indicates the potential multiple ways of SARS-CoV-2 transmission, including fecal-oral transmission, and the possibility of targeting different organs (47). Cases in adults with active virus replication in the upper respiratory tract display a shed pattern that resembles patients with influenza (48, 49). Furthermore, from biopsy samples taken from the lung, liver, and heart tissues of infected and dead adult patient, similar pathological features to SARS and MERS coronavirus infections have been found (50, 51). The lungs showed evidence of acute respiratory distress syndrome (ARDS), while the liver showed moderate microvascular steatosis and mild lobular and portal activity. The heart tissue was infiltrated with mononuclear inflammatory cells, without substantial damage (50). A recent study also found highly expressed ACE2 in proximal and distal enterocytes (52). In human small intestinal organoids (hSIOs), enterocytes were readily infected by SARS-CoV-2 (53). These all reflect the complexity of this novel virus, and we still need more data on transmission dynamics and pathology in neonates and children to further explain the virologic characteristics.

## COVID-19 in Pregnant Women and Neonates

### Pregnant Women

During the rapid spread of COVID-19 in China and other countries, SARS-CoV-2 infection in pregnant women seems inevitable. However, there are only several reports of infection in pregnant women and of neonates born to infected mothers in China. Of the 34 pregnant women who were confirmed with the SARS-CoV-2 infection in multiple hospitals in Wuhan, including one pregnant woman with a negative nucleic acid test result, 30 had a fever and 16 had a cough (54–57). Other symptoms included diarrhea in eight patients, myalgia in seven, fatigue in six, sore throat in five, shortness of breath in five, chest pain in three, headache in three, and rashes in two (54–57). Among them, 30 were in their third trimester and the other four were in the second trimester. Fetal distress was monitored in eight of the pregnant women. One case had vaginal bleeding during the third trimester, and six had premature rupture of membranes (PROM). In addition, one patient had gestational hypertension and another had preeclampsia (55). Other comorbidities included hypothyroidism and polycystic ovary syndrome (57). All patients had an epidemiological history and had been exposed to COVID-19. Most patients showed typical features of chest CT images, such as multiple plaque-like ground glass shadows in the lungs, plaque consolidation, and blurred borders (54, 55). Finally, 26 of the pregnant women delivered their babies by cesarean section, and three of them delivered vaginally. One case with a gestational age of 28 weeks had a benign outcome and did not give birth, with conserved treatment to prolong gestation (56). Furthermore, there was one miscarriage at 26 gestational weeks within the onset of bipolar disorder, and the woman required the termination of her pregnancy. It was unknown whether the outbreak of COVID-19 influenced her onset of bipolar disorder. Noticeably, four of these 34 patients developed severe pneumonia, in which one developed worse and was transferred into ICU (55, 56). The clinical characteristics of COVID-19 in pregnant women appear to be similar to those reported in non-pregnant adult patients with COVID-19, which could be further confirmed with recent cases outside Wuhan (55, 58–61). According to the recent report of 118 pregnant women with COVID-19 in Wuhan, the risk of severe disease compared favorably with the risk in the general population of patients in mainland China (62). No maternal death has been reported. Comparably, the clinical outcome of pregnant women during SARS in Hong Kong was worse than that of infected women who were not pregnant (63–65). Pregnant women infected with MERS-CoV might also develop serious diseases, and even the maternal outcome was fatal (66). Considering the relationship between SARS-CoV-2 and SARS-CoV or MERS-CoV, more cases need to be observed, and COVID-19 in perinatal pregnant women needs treatment with more caution.

### Neonates Born to Mothers With COVID-19

Of the 30 pregnant women in the third trimester mentioned above, 29 of them gave birth to 30 babies, including one set of twins (54–57). Of these, 12 were premature infants (gestational age ranging from 31 weeks to 36 weeks plus 3 days), among them three were low-birth-weight infants, and two were small-for-gestational-age (SGA) infants (54–56). The 1- and 5-min Apgar scores of all live births were 8–10, except for one LGA infant who had a 1-min Apgar score of 7- and a 5-min Apgar score of 8. Pharyngeal swab specimens were collected from 22 of the 30 neonates, and only one was positive at 36 h after birth (54–57). Six of the newborns developed shortness of breath, in which five were premature and intrauterine fetal distress was found in mothers of four neonates, but no severe neonatal asphyxia was observed. Other symptoms included vomiting, moaning, edema and skin damage, fever, milk rejection, and gastrointestinal bleeding (54). The newborn with positive SARS-CoV-2 had no fever and cough, with only mild shortness of breath (23, 57). So far, three patients developed disseminated intravascular coagulation (DIC) in two case series, possibly because of immature immune function of the neonates and suspected sepsis (26, 54). One of them eventually died, one improved with antibiotic treatment, and the other also improved after receiving intravenous immunoglobulins (IVIG) transfusion (26, 54). It suggests that gamma-globulin may be effective in severe cases. However, the dose of IVIG was not mentioned in the case and needed further exploration (54). Radiographic findings were non-specific. Within the 33 neonates born to affected mothers reported recently, chest radiographic images in the three with positive SARS-CoV-2 showed pneumonia (26). Recently, another case of neonatal death within 2 hours of birth was reported because of severe neonatal asphyxia. The mother developed severe pneumonia and septic shock after admission (60). Therefore, the severity of neonatal symptoms is closely related to the maternal condition (54). Moreover, maternal chronic illness or complications and effective treatment of the newborns may also affect their outcome (58).

However, there is no evidence that the emergence of COVID-19 in the third trimester of pregnancy may result in severe adverse outcomes in neonates, which is caused by vertical transmission in the womb (55). Amniotic fluid, umbilical cord blood, neonatal throat swabs, and even breast milk samples were collected and tested, but no SARS-CoV-2 was found (55). Pathological analysis has also showed no evidence of viral infection or chorioamnionitis in placental tissue (67). In addition, one study used public single-cell RNA sequencing databases to analyze mRNA expression profiles and found that the expression of ACE2 in different cell types in the early maternal-fetal interface was very low, which may provide an explanation of low risk of vertical transmission in COVID-19 and SARS (68). However, at least five neonates born to COVID-19 pregnant women tested positive for SARS-CoV-2 (23, 26, 27). Three infants born to mothers with COVID-19 had elevated IgM antibodies to SARS-CoV-2 (29, 30). They were delivered in negative-pressure isolation rooms, and the mothers wore masks in delivery. These results remind us that more evidence is still needed to evaluate whether vertical transmission could be a possible way of coronavirus transmission (58, 63).

### Neonatal COVID-19 From the Community

In addition, a neonate was diagnosed as having COVID-19 17 days after birth and he had a history of close contact with two confirmed cases (parents of the newborn) (22, 58). The patient's early clinical symptoms were mild, such as transient fever and diarrhea, without any severe complications. X-ray imaging of the lungs showed inflammatory changes. Repeated positive nucleic acid test results of pharyngeal and anal swabs indicated that the virus could appear in the respiratory and digestive tracts of newborns (22). This case also indicates that there is a possibility that family members or the community may be a source of neonatal infection. Another case recently reported was a 19 day-old baby boy, who also showed gastrointestinal symptoms (28). Although the symptoms could be mild, protection of the newborns still needs to be strengthened. They may show different symptoms from adults, therefore, either the parents or the doctors should be more aware of any abnormal conditions when breastfeeding.

Additionally, no cases of SARS-CoV-2 infection have been reported in women in the first trimester of pregnancy. Given that the fetus of a mother infected with SARS-CoV in the first trimester of pregnancy would develop intrauterine growth restriction (IUGR), more attention should be paid on the prevention of COVID-19 in the first trimester of pregnancy (63).

## Clinical, Radiological, and Laboratory Features in Infants and Children

The proportion of infants and children diagnosed with COVID-19 is currently small, which may be related to the lack of pathogen detection among them. It may be because they have a lower risk of exposure, or that they either have mild symptoms or are asymptomatic, which is not easily identified, rather than them being less susceptible than adults (16, 25, 69). The early stages of the COVID-19 epidemic mainly involve adults over the age of 15, indicating confirmed childhood cases are more likely transmitted from family members or the community (19). In addition, the ability of children to transmit the virus may be limited, and no clear report has been found that children can be the source of infection in adults (70).

### Clinical Features

The most common symptoms of COVID-19 in children were a fever and cough. Other symptoms included fatigue, myalgia, nausea, vomiting, and diarrhea, which seems to be milder than adults with COVID-19 (Table 1) (20, 25, 81, 82). Within 2,135 pediatric patients <18-years old who reported with COVID-19, groups of all ages were susceptible (16). The median age of all patients was 7-years, and no statistically significant difference was shown in gender (16). Among both confirmed and suspected cases, 94 (4.4%), 1088 (51.0%), and 826 (38.7%) were diagnosed as asymptomatic, mild, or moderate, respectively (16). Another report in 171 children with SARS-CoV-2 infection showed the median age was 6.7-years (25). Fever was present in 41.5% of the children at any time of the illness (25).

**Table 1:** Epidemiologic and clinical characteristics in pediatric series of COVID-19.

**Characteristic**	**Neonates** **(0–28 d)**	**Infants** **(28 d−1 y)**	**Children^**^&^**^** **(1 y−18 y)**
**Case number (*****n*****)**	7	16	44
**Sex**			
Male	7	3	22
Female	0	13	22
**Frequency of symptoms**			
Fever	4	7	31
Cough	2	8	21
Nausea/vomiting	4	2	8
Diarrhea	2	0	6
Milk rejection	1	0	0
Feeding intolerance	1	0	0
Sneezing	0	1	4
Stuffy nose	0	1	4
Running nose	0	4	2
Fatigue/malaise	0	0	1
Lethargy	1	0	0
Sore throat	0	0	10
Shortness of breath	1	2	8
None	1	1	9
**Case severity**^**^&&^**^			
Mild	2	0^*^	3
Moderate	4	5^*^	33
Severe	0	1^*^	4
Critical	1	1^*^	4
**Epidemiologic history**			
Linkage to Wuhan^**^	6	14	30^*^
Contact with infected family member	2	15^*^	16^*^
**Complication**	1	3	3
**Radiographic evidence**	5	6^*^	28^*^
**Positive nucleic acid test**			
Pharyngeal swab	7	16	43
Stool/anal swab	5^*^	4^*^	5^*^
Sputum	NA	NA	1^*^
Urine	NA	0^*^	0^*^
Blood	NA	0^*^	0^*^
**Treatment**			
Oxygen therapy	1^*^	3	6^*^
Antiviral therapy	1^*^	5^*^	19^*^
Use of corticosteroid	0^*^	1^*^	9^*^
Antibiotic therapy	2^*^	4^*^	16^*^
**Outcome**			
Discharge	6	13	27
In hospital	1	3	17
Died	0	0	0
**References**	(22, 23, 26–28)	(15, 18, 71–74)	(6, 14, 18, 21, 73–79)

*COVID-19, 2019 novel coronavirus disease. NA, not available*.

* *Some data may not be available in some cases and are not counted in the table as well*.

** *Residing in or visiting Wuhan or contact with visitors from Wuhan ≤ 2 weeks before the onset of infection*.

& *We define the age of children from 1 to 18-years old, including 1-year old*.

&& *Definitions of clinical types of COVID-19 in pediatric patients: (80)*.

*Mild disease: Mild upper respiratory symptoms for a short duration, without abnormal radiographic presentation*.

*Moderate disease: Mild pneumonia or asymptomatic with radiographic evidence*.

*Severe disease: Rapid breath (≥70 breaths per min for infants aged <1-year; ≥50 breaths per min for children aged >1-year); Hypoxia; Difficulty in feeding with dehydration; Lack of consciousness, depression, coma, convulsions*.

*Critical illness: Respiratory failure with need for mechanical ventilation; Septic shock; Organ failure that needs monitoring in the ICU*.

Specifically, symptoms could be mild in infants (28 days to 1-year), with only fever or mild upper respiratory symptoms (15, 71). However, the proportion of severe and critical cases amongst pediatric groups was highest in infants <1-year old, which reveals that young children, particularly infants, were vulnerable to SARS-CoV-2 infection (16). According to the case of a 55 day-old female infant, multiple organ damage affecting the lungs, liver, and heart may be present (72). Both the nasopharyngeal swab and stool specimen tested positive for SARS-CoV-2. The symptoms were initially mild but progressed rapidly later. Therefore, frequent and careful monitoring, as well as timely and appropriate treatment, are important in infant cases (72).

Similarly, children with SARS-CoV-2 infection may also have severe symptoms. The first severe case of childhood infection was reported on January 27, 2020, in Wuhan (21). He was a 13-month-old child, with frequent vomiting and diarrhea at first, which rapidly progressed to other acute symptoms including shortness of breath and oliguria 6 days later, which turned to ARDS, septic shock, and acute renal failure at last. He had no comorbidities. Nucleic acid tests were not positive until it was performed for the third time. Given that his immune system may be overreacted, and it was necessary to maintain acid-base balance and improve organ function in the critically ill patient, continuous renal replacement therapy (CRRT) was used and finally improved his symptoms. In severe or critically ill pediatric patients, the most common symptom is shortness of breath, and invasive mechanical ventilation may be indicated for effective respiratory support (73). Children with cancer could also be exposed to SARS-CoV-2 infection. An 8-year-old boy with acute lymphoblastic leukemia was confirmed with COVID-19 recently (73). The symptoms included pancytopenia and fever. The conditions turned critical regardless of assisted ventilation. Therefore, development of standardized guidance for prevention in children with cancer and collaboration among the pediatric oncology community are urgently required (83).

In addition, another situation also needs to be paid attention to. This was a case of a child diagnosed with COVID-19 with acute appendicitis (75). He was initially prepared for abdominal surgery for “acute appendicitis,” but he developed a fever before the operation. His mother told the doctor that he had dinner with his grandmother before, who was earlier confirmed to be SARS-CoV-2 positive. Therefore, it has to be considered that children and infants may not cooperate with the examination, and the description may be unclear. Respiratory symptoms and physical signs are not obvious among them as well. When the emergence of surgical related symptoms happens, such as acute abdominal pain as the first manifestation, the possibility of SARS-CoV-2 infection needs to be discussed, and more concern is also needed on the reasonable arrangements for surgical operations during the epidemic.

### Radiological and Laboratory Features

In addition to atypical clinical symptoms, early radiographic findings of children with pulmonary infections were also milder than those of adults, and most were nodular ground-glass changes or unilateral patchy lesions (18, 25, 76, 84). The CT characteristics were atypical, with a more localized ground glass opacity (GGO) extent, lower GGO attenuation, and relatively rare interlobular septal thickening (85). Furthermore, the CT imaging of severe cases of COVID-19 may be similar to the findings of adults, such as pulmonary parenchymal ground-glass lesions and consolidative pulmonary opacities in the lung (21, 86, 87).

On the other hand, laboratory tests of the 13-month-old severe case mentioned above showed similar characteristics to adult cases. In the acute phase of the disease, C-reactive protein was significantly increased, CD3^+^ T cells and natural killer cells were significantly reduced, and C3 and C4 levels were also significantly reduced (21). The child's T cell activation was inhibited, but the body's immune system can be over-activated, indicating the complex role of the immune system in the progression of COVID-19. Other abnormal laboratory findings in common and severe cases are elevated creatine kinase MB, decreased lymphocytes, and elevated procalcitonin and alanine aminotransferase, which indicates possible damage of multiple organs (20, 88). Noticeably, the older children may have significantly decreased lymphocytes, elevated procalcitonin, and decreased creatine kinase compared with the younger patients, such as children under 5-years old (20).

## Diagnosis and Management

### Diagnostic Methods and Differential Diagnosis

The reliability of real-time reverse transcription PCR (RT-PCR) for the detection of SARS-CoV-2 has been demonstrated, particularly in collected patient saliva or pharyngeal swabs (89, 90). Recommendations from China for the diagnosis of COVID-19 also suggest the use of real-time fluorescent RT-PCR to detect SARS-CoV-2 nucleic acid (80, 91–93). It is important especially in children with atypical symptoms (6). Another method suggested is metagenomic next-generation sequencing (mNGS) of RNA extracted from bronchoalveolar lavage fluid (BALF) or other specimens (80, 94).

However, it has to be mentioned that the first two pharyngeal swab nucleic acid tests of the severe 13-month-old child above-mentioned were negative, and they were not positive until the third nucleic acid test on the 13th day of onset (21). The delay in diagnosis and treatment of children may be fatal, since there have been two deaths in children (16, 25). Therefore, other samples should be actively explored and evaluated for the diagnostic value of SARS-CoV-2 infection in children as in recommendations, such as the upper or lower respiratory tract, blood, stool, and urine, in order to increase the positive rate of nucleic acid detection (80, 92, 93, 95). Given that neonates seem to manifest gastrointestinal symptoms more commonly, persistent anal swab tests might be more useful (22, 28, 52).

However, there are still some atypical cases with epidemiological history, respiratory or gastrointestinal symptoms, and positive chest CT manifestations that may have negative RT-PCR results for SARS-CoV-2 in adults (96–98). In the diagnosis of patients with suspected COVID-19, the positive rate of chest CT imaging may be even higher than that of RT-PCR analysis. The patients may first show a positive chest CT, and the improvement of the chest CT can be reflected earlier in recovery, indicating its better sensitivity in diagnosis of COVID-19 (99). Given that chest radiographic images could also reflect abnormalities in most cases of neonates and children, the combination of imaging and nucleic acid tests may be a better method for comprehensive evaluation of pediatric patients with COVID-19 (25, 26). Additionally, chest X-rays and CTs should be performed with more caution in pediatric patients for protection to this vulnerable population from the risk of radiation (85). Moreover, application of pulmonary ultrasounds in neonates may show pulmonary abnormalities of COVID-19 with better sensitivity and safety than chest X-rays and CTs, which provides more chances in monitoring and evaluation of the disease (100).

In addition, specific antibody tests are available for retrospective diagnostic and epidemiological studies, which have already been used as one of the methods for diagnosis of COVID-19 according to the latest version of New Coronavirus Pneumonia Prevention and Control Protocol from National Health Commission of the People's Republic of China (China NHC) (80). IgM antibodies to SARS-CoV-2 in neonates may also have indication in vertical transmission (29, 30). Recently, a new platform called Cas13-assisted viral expression and read restriction (CARVER) was developed for rapid diagnosis of ssRNA viruses. It mainly uses Cas13 to detect and destroy viral RNA (101). The CRISPR system seems to illustrate the unique and comprehensive prospect of virus infection diagnosis and treatment in future (101, 102).

Finally, the additive effect of seasonal influenza on the COVID-19 epidemic may interfere with doctors' clinical decisions, so more tests should be considered to distinguish COVID-19 from other acute respiratory infections with similar symptoms in order to strengthen management of COVID-19 (77).

### Management

**Figure 1:**
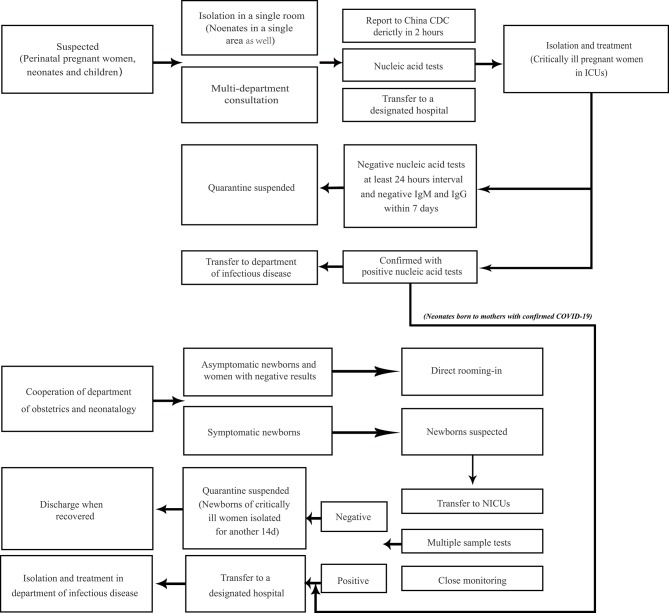
Management plan for prevention and control of COVID-19 in perinatal pregnant women, neonates, and children. Neonates and children of high risk need medical observation for 14 days, which can be terminated in advance based on epidemiology history, clinical characteristics, and laboratory tests. Neonates from women with confirmed COVID-19 should be treated according to the management of neonatal confirmed case, although they may not be infected. Perinatal pregnant women refer to women in peripartum. Pregnant women at birth with suspected infection follow the diagram below. Neonates with epidemiology history such as being born to SARS-CoV-2 infected mothers within 14 days before and 28 days after delivery, or direct exposure to family members, caregivers, medical staff, or visitors with COVID-19 should be suspected with infection, whether with or without symptoms. Suspected cases with both negative nucleic acid tests at least 24 h interval and negative IgM and IgG to SARS-CoV-2 within 7 days will be suspended quarantine. The management plan in perinatal pregnant women, neonates, and children from the community is from the latest New Coronavirus Pneumonia Prevention and Control Protocol from China NHC (80). The management plan in neonates born to the mothers is from the national guideline of perinatal and neonatal management plan of SARS-CoV-2 infection (92).

In the prevention and management of COVID-19, pregnant women, neonates, and children should be considered as the main high-risk population (58). China NHC has provided prevention and control protocols for COVID-19 and updated these during the epidemic. The latest 7th version provided on March 3 covered all populations in China (80). Furthermore, specific recommendations for neonates and children were also provided as national consensus guidelines (91–93). The guidelines define the suspected and confirmed cases in different populations, as well as the criteria for discharge (80, 92, 93). Figure 1 is extracted from these guidelines as a concise protocol for management in pregnant women, neonates, and children. According to the management plan in pregnant women and neonates, newborns of mothers suspected or diagnosed with SARS-CoV-2 infection in delivery should be well-rescued and cared for via the cooperation of the department of obstetrics and neonatology (92). All neonates with suspected or laboratory-confirmed COVID-19 should be admitted to neonatal intensive care units (NICUs) (91, 92). High-risk neonates should be placed in a designated room for medical observation for at least 14 days (91). If a pregnant woman or newborn is diagnosed or suspected of infection, breastfeeding should be avoided (91). Recently, a global guideline for pregnant women with suspected SARS-CoV-2 infection has also been provided (103). Moreover, recent research found that there may be potential risks of SARS-CoV-2 transmission in hospital settings, hence pediatricians and neonatologists should be more careful in treating the patients in NICUs and pediatric intensive care units (PICUs) (104, 105). Home confinement and online courses may have a psychological impact on children and adolescents, emphasizing the importance of the awareness and guidelines provided for students from the government (36, 106). Finally, it must be noted that RT-PCR-positive results may still be found in pediatric patients recovered from COVID-19 (47, 71). In infants and young children, negative pharyngeal swab results may have already been detected, but viral nucleic acid can still be detected in fecal specimens (78). A contingency plan for NICUs recently suggested SARS-CoV-2 negative results of respiratory specimens or anal swabs should be obtained at least 48 h before discharge (91). Further isolation and long-term follow-up of discharged children with positive results of anal swabs should be considered for their potential transmission in public health (107).

## Treatment and Outcome

### Treatment

The treatment of neonates and children is similar to that of adults, but it also has its own characteristics. To date, there are no specific drugs that can cure COVID-19, and vaccines are still being studied. The purpose of treatment is to improve the patient's symptoms and provide better support. The most effective treatment is oxygen therapy, which is important in treating symptomatic newborns and critically ill children. It is closely related to the children's final outcome, and early treatment can reduce complications, such as ARDS or respiratory failure (108). In adult COVID-19 cases, severe ARDS is always associated with high mortality (109). Therefore, timely ventilation might be vital in preventing ARDS or respiratory failure in pediatric COVID-19 cases.

Secondly, the effect of antiviral treatment in COVID-19 is still uncertain. The first reported case in the United States benefited from an investigational antiviral drug called remdesivir, which has also proved to have a clinical benefit in the rhesus macaque model of MERS-CoV infection (110, 111). Lopinavir–ritonavir treatment reported no benefit in adult severe cases (112). In 36 pediatric cases, mild cases received interferon alfa by aerosolization twice a day, while most moderate cases were given interferon alfa with lopinavir/ritonavir syrup twice a day (20). However, no specific improvement of such antiviral treatment has been analyzed in pediatric cases, and it would be helpful to provide more clinical trials in the future.

In addition, the use of corticosteroids remains controversial. WHO's current interim guidelines recommended against the use of corticosteroids unless indicated for another reason (113). Different studies have shown that it could be either beneficial or unfavorable for patients with coronavirus infection (such as SARS and MERS) (114, 115). Recently, expert consensus in China has advised against the use of corticosteroids in children under 18-years-old (116).

Moreover, traditional Chinese medicine may have a therapeutic effect on COVID-19, but it is not fully recommended for children as well, because childhood toxicity is uncertain (117, 118). Intravenous immunoglobulin (IVIG) is used to rescue newborns and critically ill children and may improve the disease (21, 54). Finally, recent studies on the structure of SARS-CoV-2 spike glycoprotein and cell entry have provided possible solutions for vaccine design and the application of protease inhibitors (119, 120). The blocking effect of cross-neutralizing antibodies may also indicate the feasibility of developing convalescent plasma therapy from healthy donors as a clinical trial in China, which has already been used in severe and critically ill pediatric and adult cases (77, 119–122).

### Outcome

In elderly patients with COVID-19 (>65-years), especially those with comorbidities, clinical outcomes are usually poor (123). However, to date, only two neonates born to mother with COVID-19 and two children with COVID-19 have been reported to have died in China, and most newborns and children have eventually recovered. Some patients were still isolated in hospital for further observation (81). Further analysis is needed to better understand the prognosis of COVID-19 in neonates and children. Neonates born to mothers with COVID-19 in the first and second trimester need close monitoring and further assessment. In addition, follow-up studies have shown that some children with SARS had deficiencies in lung function assessment and decreased exercise capacity (124). Therefore, we call for long-term follow-up and comprehensive assessment of infected newborns and children after discharge to determine the prognosis of COVID-19.

## Conclusions

Since 2003, the Chinese government has gained many lessons from the SARS outbreak. In the COVID-19 epidemic, besides China, the global response has been more timely, including coordination among different countries, sharing of disease information and cases, government and media reports, and public response (24, 125). The Chinese government has taken effective measures to control the epidemic. The experts also made recommendations for high-risk groups including pregnant women, newborns, and children. In addition, compared to adults, children have milder conditions, a faster recovery, and a better prognosis (126). A series of improvements to date have been applied to prevent the prevalence of COVID-19 in the global community. However, given that the symptoms of COVID-19 in neonates and children are atypical, and transmission within family members is quite common, more effort should be made to protect this high-risk population. Although there is still no direct evidence of vertical transmission, the rescue of newborns of infected pregnant women in delivery should not be delayed. Furthermore, development of vaccines and effective treatments like novel antiviral drugs is also urgent and necessary. Current outbreak will be restricted only if the whole world stands together and cooperates constantly.

## Author Contributions

YY and PC contributed to the conception of the review. YY contributed to the literature search and writing of the manuscript. Final integration and editing were done by PC. The table and figure were drafted by YY.

## Conflict of Interest

The authors declare that the research was conducted in the absence of any commercial or financial relationships that could be construed as a potential conflict of interest.

## Abbreviations

2019-nCoV: 2019 Novel coronavirus
ACE2: angiotensin-converting enzyme 2
ARDS: acute respiratory distress syndrome
BALF: bronchoalveolar lavage fluid
CARVER: Cas13-assisted viral expression and read restriction
China CDC: Chinese Center for Disease Control and Prevention
China NHC: National Health Commission of the People's Republic of China
CoVs: Coronaviruses
COVID-19: Coronavirus Disease 2019
DIC: disseminated intravascular coagulation
hSIOs: human small intestinal organoids
IUGR: intrauterine growth restriction
IVIG: intravenous immunoglobulin
MERS: Middle East respiratory syndrome
MERS-CoV: Middle East Respiratory Syndrome Coronavirus
mNGS: metagenomic next-generation sequencing
NRDS: neonatal respiratory distress syndrome
NICUs: neonatal intensive care units
PHEIC: public health emergency of international concern
PICUs: pediatric intensive care units
PROM: premature rupture of membranes
S: spikes
SARS: severe acute respiratory syndrome
SARS-CoV-2: Severe Acute Respiratory Syndrome Coronavirus-2 wryip
SGA: small-for-gestational-age
ssRNA: single-stranded sense RNA
rRT-PCR: real-time reverse transcription PCR
WHO: World Health Organization.

## References

[1] Ksiazek TG Erdman D Goldsmith CS Zaki SR Peret T Emery S. A novel coronavirus associated with severe acute respiratory syndrome. N Engl J Med. (2003) 348:1953–66. 10.1056/NEJMoa030781 12690092

[2] Zumla A Hui DS Perlman S. Middle East respiratory syndrome. Lancet. (2015) 386:995–1007. 10.1016/S0140-6736(15)60454-8 26049252 PMC4721578

[3] Wu F Zhao S Yu B Chen Y-M Wang W Song Z-G. A new coronavirus associated with human respiratory disease in China. Nature. (2020) 579:265-9. 10.1038/s41586-020-2008-3 32015508 PMC7094943

[4] Jiang S Shi ZL. The first disease X is caused by a highly transmissible acute respiratory syndrome coronavirus. Virol Sin. (2020). 10.1007/s12250-020-00206-5. [Epub ahead of print]. 32060789 PMC7091198

[5] Zhu N Zhang D Wang W Li X Yang B Song J. A novel coronavirus from patients with Pneumonia in China, 2019. N Engl J Med. (2020) 382:727–33. 10.1056/NEJMoa2001017 31978945 PMC7092803

[6] Chan JF Yuan S Kok KH To KK Chu H Yang J. A familial cluster of pneumonia associated with the 2019 novel coronavirus indicating person-to-person transmission: a study of a family cluster. Lancet. (2020) 395:514–23. 10.1016/S0140-6736(20)30154-9 31986261 PMC7159286

[7] Chang D Lin M Wei L Zhu G Dela Cruz CS Sharma L Epidemiologic and clinical characteristics of novel coronavirus infections involving 13 patients outside Wuhan, China. JAMA. (2020) 323:1092–3. 10.1001/jama.2020.1623 PMC7042871 32031568

[8] Backer JA Klinkenberg D Wallinga J Incubation period of 2019 novel coronavirus (2019-nCoV) infections among travellers from Wuhan, China, 20-28 January *Euro Surveill*. (2020) 25:2000062 10.2807/1560-7917.ES.2020.25.5.2000062 PMC7014672 32046819

[9] Yu P Zhu J Zhang Z Han Y Huang L A familial cluster of infection associated with the 2019 novel coronavirus indicating potential person-to-person transmission during the incubation period. J Infect Dis. (2020) 221:1757–61. 10.1093/infdis/jiaa077 32067043 PMC7107453

[10] Hoehl S Berger A Kortenbusch M Cinatl J Bojkova D Behrens P. Evidence of SARS-CoV-2 Infection in Returning Travelers from Wuhan, China. N Engl J Med. (2020) 382:1278–80. 10.1056/NEJMc2001899 32069388 PMC7121749

[11] Bai Y Yao L Wei T Tian F Jin D-Y Chen L Presumed asymptomatic carrier transmission of COVID-19. JAMA. (2020) 323:1406–7. 10.1001/jama.2020.2565 PMC7042844 32083643

[12] WHO Statement on the Second Meeting of the International Health Regulations (2005) Emergency Committee Regarding the Outbreak of Novel Coronavirus (2019-nCoV). (2020). Available online at: https://www.who.int/news-room/detail/30-01-2020-statement-on-the-second-meeting-of-the-international-health-regulations-(2005)-emergency-committee-regarding-the-outbreak-of-novel-coronavirus-(2019-ncov) (accessed Mar 3, 2020).

[13] WHO WHO Director-General's Opening Remarks at the Media Briefing on COVID-19 - 11 March. (2020). Available online at: https://www.who.int/dg/speeches/detail/who-director-general-s-opening-remarks-at-the-media-briefing-on-covid-19–11-march-2020 (accessed Apr 6, 2020).

[14] Cai JH Wang XS Ge YL Xia AM Chang HL Tian H [First case of 2019 novel coronavirus infection in children in Shanghai]. China J Pediatr. (2020) 58:E002 10.3760/cma.j.issn.0578-1310.2020.0002 32023679

[15] Wei M Yuan J Liu Y Fu T Yu X Zhang ZJ Novel coronavirus infection in hospitalized infants under 1 year of age in China. JAMA. (2020) 323:1313–4. 10.1001/jama.2020.2131 PMC7042807 32058570

[16] Dong Y Mo X Hu Y Qi X Jiang F Jiang Z Epidemiology of COVID-19 Among Children in China. Pediatrics. (2020) 145:e20200702 10.1542/peds.2020-0702 32179660

[17] Hon KL Leung CW Cheng WT Chan PK Chu WC Kwan YW. Clinical presentations and outcome of severe acute respiratory syndrome in children. Lancet. (2003) 361:1701–3. 10.1016/S0140-6736(03)13364-8 12767737 PMC7112484

[18] Cai J Xu J Lin D Yang Z Xu L Qu Z. A case series of children with 2019 novel coronavirus infection: clinical and epidemiological features. Clin Infect Dis. (2020). 10.1093/cid/ciaa198. [Epub ahead of print]. 32112072 PMC7108143

[19] Li Q Guan X Wu P Wang X Zhou L Tong Y. Early transmission dynamics in Wuhan, China, of novel coronavirus-infected Pneumonia. N Engl J Med. (2020) 382:1199–207. 10.1056/NEJMoa2001316 31995857 PMC7121484

[20] Qiu H Wu J Hong L Luo Y Song Q Chen D. Clinical and epidemiological features of 36 children with coronavirus disease 2019. (COVID-19) in Zhejiang, China: an observational cohort study. Lancet Infect Dis. (2020). 10.1016/S1473-3099(20)30198-5. [Epub ahead of print]. 32220650 PMC7158906

[21] Chen F Liu ZS Zhang FR Xiong RH Chen Y Cheng XF [First case of severe childhood novel coronavirus pneumonia in China]. Zhonghua Er Ke Za Zhi. (2020) 58:179–82. 10.3760/cma.j.issn.0578-1310.2020.0005 32135586

[22] Zeng LK Tao XW Yuan WH Liu X Liu ZS. [First case of neonate infected with novel coronavirus pneumonia in China]. Zhonghua Er Ke Za Zhi. (2020) 58:E009. 10.3760/cma.j.issn.0578-1310.2020.0009 32065520

[23] Wang S Guo L Chen L Liu W Cao Y Zhang Y A case report of neonatal COVID-19 infection in China. Clin Infect Dis. (2020) 12:ciaa225 10.1093/cid/ciaa225 PMC7108144 32161941

[24] Wu Z McGoogan JM Characteristics of and important lessons from the coronavirus disease 2019 (COVID-19) outbreak in China: summary of a report of 72 314 cases from the Chinese center for disease control and prevention. JAMA. (2020) 323:1239–42. 10.1001/jama.2020.2648 32091533

[25] Lu X Zhang L Du H Zhang J Li YY Qu J. SARS-CoV-2 infection in children. N Engl J Med. (2020) 382:1663–5. 10.1056/NEJMc2005073 32187458 PMC7121177

[26] Zeng L Xia S Yuan W Yan K Xiao F Shao J. Neonatal early-onset infection with SARS-CoV-2 in 33 neonates born to mothers with COVID-19 in Wuhan, China. JAMA Pediatr. (2020). 10.1001/jamapediatrics.2020.0878. [Epub ahead of print]. 32215598 PMC7099530

[27] Li M Xu M Zhan W Han T Zhang G Lu Y [Report of the first cases of mother and infant infections with 2019 novel coronavirus in Xinyang City Henan Province]. Chin J Infect Dis. (2020) 38:E007 10.3760/cma.j.issn.1000-6680.2020.0007

[28] Wang J Wang D Chen GC Tao XW Zeng LK. [SARS-CoV-2 infection with gastrointestinal symptoms as the first manifestation in a neonate]. Zhongguo Dang Dai Er Ke Za Zhi. (2020) 22:211–4. 10.7499/j.issn.1008-8830.2020.03.006 32204755 PMC7389603

[29] Zeng H Xu C Fan J Tang Y Deng Q Zhang W Antibodies in infants born to mothers with COVID-19 Pneumonia. JAMA. (2020) 323:1848-9. 10.1001/jama.2020.4861. PMC7099444 32215589

[30] Dong L Tian J He S Zhu C Wang J Liu C Possible vertical transmission of SARS-CoV-2 from an infected mother to her newborn. JAMA. (2020) 323:1846-8. 10.1001/jama.2020.4621. PMC7099527 32215581

[31] Riou J Althaus CL. Pattern of early human-to-human transmission of Wuhan 2019 novel coronavirus (2019-nCoV), December 2019 to January 2020. Euro Surveill. (2020) 25:2000058. 10.2807/1560-7917.ES.2020.25.4.2000058 32019669 PMC7001239

[32] Wu JT Leung K Leung GM. Nowcasting and forecasting the potential domestic and international spread of the 2019-nCoV outbreak originating in Wuhan, China: a modelling study. Lancet. (2020) 395:689–97. 10.1016/S0140-6736(20)30260-9 32014114 PMC7159271

[33] Zhou T Liu Q Yang Z Liao J Yang K Bai W. Preliminary prediction of the basic reproduction number of the Wuhan novel coronavirus 2019-nCoV. J Evid Based Med. (2020) 13:3–7. 10.1111/jebm.12376 32048815 PMC7167008

[34] Tang B Wang X Li Q Bragazzi NL Tang S Xiao Y. Estimation of the transmission risk of the 2019-nCoV and its implication for public health interventions. J Clin Med. (2020) 9:E462. 10.2139/ssrn.3525558 32046137 PMC7074281

[35] Wang G Jin X. The progress of 2019 novel coronavirus event in China. J Med Virol. (2020) 92:468–72. 10.1002/jmv.25705 32048741 PMC7166326

[36] Wang G Zhang Y Zhao J Zhang J Jiang F. Mitigate the effects of home confinement on children during the COVID-19 outbreak. Lancet. (2020) 395:945–7. 10.1016/S0140-6736(20)30547-X 32145186 PMC7124694

[37] Patel A Jernigan DB. Initial public health response and interim clinical guidance for the 2019 novel coronavirus outbreak - United States, December 31, 2019-February 4, 2020. MMWR Morb Mortal Wkly Rep. (2020) 69:140–6. 10.15585/mmwr.mm6908e1 32027631 PMC7004396

[38] Gostin LO Hodge JG Jr. US emergency legal responses to novel coronavirus: balancing public health and civil liberties. JAMA. (2020) 323:1131–2. 10.1001/jama.2020.2025 32207808

[39] Chen Y Liu Q Guo D. Emerging coronaviruses: genome structure, replication, and pathogenesis. J Med Virol. (2020) 92:418–23. 10.1002/jmv.25681 31967327 PMC7167049

[40] Lu R Zhao X Li J Niu P Yang B Wu H. Genomic characterisation and epidemiology of 2019 novel coronavirus: implications for virus origins and receptor binding. Lancet. (2020) 395:565–74. 10.1016/S0140-6736(20)30251-8 32007145 PMC7159086

[41] Zhou P Yang XL Wang XG Hu B Zhang L Zhang W. A pneumonia outbreak associated with a new coronavirus of probable bat origin. Nature. (2020) 579:270–3. 10.1038/s41586-020-2012-7 32015507 PMC7095418

[42] Chan JF Kok KH Zhu Z Chu H To KK Yuan S. Genomic characterization of the 2019 novel human-pathogenic coronavirus isolated from a patient with atypical pneumonia after visiting Wuhan. Emerg Microbes Infect. (2020) 9:221–36. 10.1080/22221751.2020.1719902 31987001 PMC7067204

[43] Lam TT Shum MH Zhu HC Tong Y-G Ni X-B Liao Y-S. Identifying SARS-CoV-2 related coronaviruses in malayan pangolins. Nature. (2020). 10.1038/s41586-020-2169-0. [Epub ahead of print]. 32218527

[44] Wu A Peng Y Huang B Ding X Wang X Niu P. Genome composition and divergence of the novel coronavirus (2019-nCoV) originating in China. Cell Host Microbe. (2020) 27:325–8. 10.1016/j.chom.2020.02.001 32035028 PMC7154514

[45] Wrapp D Wang N Corbett KS Goldsmith JA Hsieh C-L Abiona O. Cryo-EM structure of the 2019-nCoV spike in the prefusion conformation. Science. (2020) 367:1260–3. 10.1101/2020.02.11.944462 32075877 PMC7164637

[46] Yan R Zhang Y Li Y Xia L Guo Y Zhou Q. Structural basis for the recognition of the SARS-CoV-2 by full-length human ACE2. Science. (2020) 367:1444–8. 10.1126/science.abb2762 32132184 PMC7164635

[47] Xu Y Li X Zhu B Fang C Gong Y Guo Q. Characteristics of pediatric SARS-CoV-2 infection and potential evidence for persistent fecal viral shedding. Nat Med. (2020) 26:502–5. 10.1038/s41591-020-0817-4 32284613 PMC7095102

[48] Wölfel R Corman VM Guggemos W Seilmaier M Zange S Müller MA. Virological assessment of hospitalized patients with COVID-2019. Nature. (2020). 10.1038/s41586-020-2196-x. [Epub ahead of print]. 32235945

[49] Zou L Ruan F Huang M Liang L Huang H Hong Z. SARS-CoV-2 viral load in upper respiratory specimens of infected patients. N Engl J Med. (2020) 382:1177–9. 10.1056/NEJMc2001737 32074444 PMC7121626

[50] Xu Z Shi L Wang Y Zhang J Huang L Zhang C. Pathological findings of COVID-19 associated with acute respiratory distress syndrome. Lancet Respir Med. (2020) 8:420–2. 10.1016/S2213-2600(20)30076-X 32085846 PMC7164771

[51] Bradley BT Bryan A. Emerging respiratory infections: the infectious disease pathology of SARS, MERS, pandemic influenza, and Legionella. Semin Diagn Pathol. (2019) 36:152–9. 10.1053/j.semdp.2019.04.006 31054790 PMC7125557

[52] Liang W Feng Z Rao S Xiao C Xue X Lin Z. Diarrhoea may be underestimated: a missing link in 2019 novel coronavirus. Gut. (2020) 69:1141–3. 10.1101/2020.02.03.20020289 32102928

[53] Lamers MM Beumer J van der Vaart J Knoops K Puschhof J Breugem TI. SARS-CoV-2 productively infects human gut enterocytes. Science. (2020). 10.1126/science.abc1669. [Epub ahead of print]. 32358202 PMC7199907

[54] Zhu H Wang L Fang C Peng S Zhang L Chang G. Clinical analysis of 10 neonates born to mothers with 2019-nCoV pneumonia. Transl Pediatr. (2020) 9:51–60. 10.21037/tp.2020.02.06 32154135 PMC7036645

[55] Chen H Guo J Wang C Luo F Yu X Zhang W. Clinical characteristics and intrauterine vertical transmission potential of COVID-19 infection in nine pregnant women: a retrospective review of medical records. Lancet. (2020) 395:809–15. 10.1016/S0140-6736(20)30360-3 32151335 PMC7159281

[56] Lei D Wang C Li C Zhou Z Liu S Rong Z [Clinical characteristics of COVID-19 in pregnancy: analysis of nine cases]. Chin J Perinat Med. (2020) 23 10.3760/cma.j.cn113903-20200216-00117

[57] Yu N Li W Kang Q Xiong Z Wang S Lin X. Clinical features and obstetric and neonatal outcomes of pregnant patients with COVID-19 in Wuhan, China: a retrospective, single-centre, descriptive study. Lancet Infect Dis. (2020) 20:559–64. 10.1016/S1473-3099(20)30176-6 32220284 PMC7158904

[58] Qiao J. What are the risks of COVID-19 infection in pregnant women? Lancet. (2020) 395:760–2. 10.1016/S0140-6736(20)30365-2 32151334 PMC7158939

[59] Wang X Zhou Z Zhang J Zhu F Tang Y Shen X. A case of 2019 Novel coronavirus in a pregnant woman with preterm delivery. Clin Infect Dis. (2020). 10.1093/cid/ciaa200. [Epub ahead of print]. 32119083 PMC7108126

[60] Yan J Guo J Fan C Juan J Yu X Li J. Coronavirus disease 2019 (COVID-19) in pregnant women: A report based on 116 cases. Am J Obstet Gynecol. (2020). 10.1016/j.ajog.2020.04.014. [Epub ahead of print]. 32335053 PMC7177142

[61] Yao L Wang J Zhao J Cui J Hu Z [Asymptomatic COVID-19 infection in pregnant woman in the third trimester: a case report]. Chin J Perinat Med. (2020) 23 10.3760/cma.j.cn113903-20200221-00143

[62] Chen L Li Q Zheng D Jiang H Wei Y Zou L. Clinical characteristics of pregnant women with covid-19 in Wuhan, China. N Engl J Med. (2020). 10.1056/NEJMc2009226. [Epub ahead of print]. 32302077 PMC7182016

[63] Schwartz DA Graham AL. Potential maternal and infant outcomes from (Wuhan) coronavirus 2019-nCoV infecting pregnant women: lessons from SARS, MERS, and other human coronavirus infections. Viruses. (2020) 12:194. 10.3390/v12020194 32050635 PMC7077337

[64] Wong SF Chow KM Leung TN Ng WF Ng TK Shek CC. Pregnancy and perinatal outcomes of women with severe acute respiratory syndrome. Am J Obstet Gynecol. (2004) 191:292–7. 10.1016/j.ajog.2003.11.019 15295381 PMC7137614

[65] Robertson CA Lowther SA Birch T Tan C Sorhage F Stockman L. SARS and pregnancy: a case report. Emerg Infect Dis. (2004) 10:345–8. 10.3201/eid1002.030736 15030710 PMC3322896

[66] Alserehi H Wali G Alshukairi A Alraddadi B. Impact of middle east respiratory syndrome coronavirus (MERS-CoV) on pregnancy and perinatal outcome. BMC Infect Dis. (2016) 16:105. 10.1186/s12879-016-1437-y 26936356 PMC4776369

[67] Chen S Huang B Luo DJ Li X Yang F Zhao Y. [Pregnant women with new coronavirus infection: a clinical characteristics and placental pathological analysis of three cases]. Zhonghua Bing Li Xue Za Zhi. (2020) 49:E005. 10.3760/cma.j.cn112151-20200225-00138 32114744

[68] Zheng QL Duan T Jin LP Single cell RNA expression profiling of ACE2 and AXL in the human maternal–fetal interface. Reprod Dev Med. (2020) 4:7–10. 10.4103/2096-2924.278679

[69] Castagnoli R Votto M Licari A Brambilla I Bruno R Perlini S. Severe acute respiratory syndrome coronavirus 2 (SARS-CoV-2) infection in children and adolescents: a systematic review. JAMA Pediatr. (2020). 10.1001/jamapediatrics.2020.1467. [Epub ahead of print]. 32320004

[70] Fang F Luo XP [Facing the pandemic of 2019 novel coronavirus infections: the pediatric perspectives]. Zhonghua Er Ke Za Zhi. (2020) 58:E001 10.3760/cma.j.issn.0578-1310.2020.0001 32023678

[71] Zhang Y Lin D Xiao M Wang JC Wei Y Lei ZX [2019-novel coronavirus infection in a three-month-old baby]. Zhonghua Er Ke Za Zhi. (2020) 58:E006 10.3760/cma.j.issn.0578-1310.2020.0006 32043842

[72] Cui Y Tian M Huang D Wang X Huang Y Fan L A 55-Day-Old female infant infected with COVID 19: presenting with pneumonia, liver injury, and heart damage. J Infect Dis. (2020) 221:1775–81. 10.1093/infdis/jiaa113 32179908 PMC7184483

[73] Sun D Li H Lu XX Xiao H Ren J Zhang F-R. Clinical features of severe pediatric patients with coronavirus disease 2019 in Wuhan: a single center's observational study. World J Pediatr. (2020). 10.1007/s12519-020-00354-4. [Epub ahead of print]. 32193831 PMC7091225

[74] Xiong D Jiang J Feng Y [Novel coronavirus pneumonia in children: a report of two cases]. Chin Pediatr Emerg Med. (2020) 10.3760/cma.j.issn.1673-4912.2020.0002. [Epub ahead of print].

[75] Wang H Duan X Yan X Sun R Liu X Ji S [A case of novel coronavirus pneumonia complicated with acute appendicitis in children]. Chin J Pediatr Surg. (2020) 41 10.3760/cma.j.cn421158-20200216-00076

[76] Feng K Yun Y Wang X Yang GD Zheng YJ Lin CM [Analysis of CT features of 15 Children with 2019 novel coronavirus infection]. Zhonghua Er Ke Za Zhi. (2020) 58:E007 10.3760/cma.j.issn.0578-1310.2020.0007 32061200

[77] Liu W Zhang Q Chen J Xiang R Song H Shu S. Detection of covid-19 in children in early January 2020 in Wuhan, China. N Engl J Med. (2020) 382:1370–1. 10.1056/NEJMc2003717 32163697 PMC7121643

[78] Zhang GX Zhang AM Huang L Cheng LY Liu ZX Peng XL. [Twin girls infected with SARS-CoV-2]. Chin J Contemp Pediatr. (2020) 22:221–5. 10.7499/j.issn.1008-8830.2020.03.008 32204757 PMC7389606

[79] Deng H Zhang Y Wang Y Li F [Novel coronavirus infection in children: a report of two cases]. Chin Pediatr Emerg Med. (2020) 27:81–3. 10.3760/cma.j.issn.1673-4912.2020.0001

[80] China NHC *New Coronavirus Pneumonia Prevention and Control Protocol*, 7th ed. National Health Commission of the People's Republic of China (2020) Available online at: http://www.nhc.gov.cn/yzygj/s7653p/202003/46c9294a7dfe4cef80dc7f5912eb1989/files/ce3e6945832a438eaae415350a8ce964.pdf (accessed Mar 3, 2020).

[81] Wang D Ju X Xie F Lu Y Li FY Huang HH. [Clinical analysis of 31 cases of 2019 novel coronavirus infection in children from six provinces (autonomous region) of northern China]. Zhonghua Er Ke Za Zhi. (2020) 58:E011. 10.3760/cma.j.cn112140-20200225-00138 32118389

[82] Su L Ma X Yu H Zhang Z Bian P Han Y. The different clinical characteristics of corona virus disease cases between children and their families in China - the character of children with COVID-19. Emerg Microbes Infect. (2020) 9:707–13. 10.1080/22221751.2020.1744483 32208917 PMC7103724

[83] Kotecha RS. Challenges posed by COVID-19 to children with cancer. Lancet Oncol. (2020) 21:e235. 10.1016/S1470-2045(20)30205-9 32220660 PMC7270527

[84] Zhou Y Yang GD Feng K Huang H Yun YX Mou XY. [Clinical features and chest CT findings of coronavirus disease 2019 in infants]. Zhongguo Dang Dai Er Ke Za Zhi. (2020) 22:215–20. 10.7499/j.issn.1008-8830.2020.03.007 32204756 PMC7389590

[85] Duan YN Zhu YQ Tang LL Qin J. CT features of novel coronavirus pneumonia (COVID-19) in children. Eur Radiol. (2020). 10.1007/s00330-020-06860-3. [Epub ahead of print]. 32291501 PMC7156230

[86] Pan Y Guan H Zhou S Wang Y Li Q Zhu T. Initial CT findings and temporal changes in patients with the novel coronavirus pneumonia (2019-nCoV): a study of 63 patients in Wuhan, China. Eur Radiol. (2020). 10.1007/s00330-020-06731-x. [Epub ahead of print]. 32055945 PMC7087663

[87] Chung M Bernheim A Mei X Zhang N Huang M Zeng X. CT Imaging features of 2019 novel coronavirus (2019-nCoV) Radiology. (2020) 295:202–7. 10.1148/radiol.2020200230 32017661 PMC7194022

[88] Ma Y Xia S Wang M Zhang S Du W Chen Q. [Clinical features of children with SARS-CoV-2 infection: an analysis of 115 cases]. Zhongguo Dang Dai Er Ke Za Zhi. (2020) 22:290–3. 10.7499/j.issn.1008-8830.2003016 32312363 PMC7389688

[89] To KK Tsang OT Chik-Yan Yip C Chan K-H Wu T-C Chan JMC. Consistent detection of 2019 novel coronavirus in saliva. Clin Infect Dis. (2020). 10.1093/cid/ciaa149. [Epub ahead of print]. 32047895 PMC7108139

[90] Corman VM Landt O Kaiser M Molenkamp R Meijer A Chu DK. Detection of 2019 novel coronavirus (2019-nCoV) by real-time RT-PCR. Euro Surveill. (2020) 25:2000045. 10.2807/1560-7917.ES.2020.25.3.2000045 31992387 PMC6988269

[91] Wang J Qi H Bao L Li F Shi Y National Clinical Research Center for Child Health and Disorders and Pediatric Committee of Medical Association of Chinese People's Liberation Army. A contingency plan for the management of the 2019 novel coronavirus outbreak in neonatal intensive care units. Lancet Child Adolesc Health. (2020) 4:258–9. 10.1016/S2352-4642(20)30040-7 32043976 PMC7128924

[92] Working Group for the Prevention and Control of Neonatal SARS-CoV-2 Infection in the Perinatal Period of the Editorial Committee of Chinese Journal of Contemporary Pediatrics [Perinatal and neonatal management plan for prevention and control of SARS-CoV-2 infection (2nd Edition)]. Zhongguo Dang Dai Er Ke Za Zhi. (2020) 22:195–8. 10.7499/j.issn.1008-8830.2020.03.003 32204752 PMC7389602

[93] Shen KL Yang YH Jiang RM Wang T-Y Zhao D-C Jiang Y. Updated diagnosis, treatment and prevention of COVID-19 in children: experts' consensus statement (condensed version of the second edition). World J Pediatr. (2020). 10.1007/s12519-020-00362-4. [Epub ahead of print]. 32333248 PMC7180653

[94] Chen L Liu W Zhang Q Xu K Ye G Wu W. RNA based mNGS approach identifies a novel human coronavirus from two individual pneumonia cases in 2019 Wuhan outbreak. Emerg Microbes Infect. (2020) 9:313–9. 10.1080/22221751.2020.1725399 32020836 PMC7033720

[95] Wang W Xu Y Gao R Lu R Han K Wu G Detection of SARS-CoV-2 in different types of clinical specimens. JAMA. (2020) 323:1843–4. 10.1001/jama.2020.3786. PMC7066521 32159775

[96] Xie X Zhong Z Zhao W Zheng C Wang F Liu J. Chest CT for typical 2019-nCoV Pneumonia: relationship to negative RT-PCR testing. Radiology. (2020). 10.1148/radiol.2020200343. [Epub ahead of print]. 32049601 PMC7233363

[97] Hao W. Clinical features of atypical 2019 novel coronavirus pneumonia with an initially negative RT-PCR assay. J Infect. (2020). 10.1016/j.jinf.2020.02.008. [Epub ahead of print]. 32092387 PMC7126654

[98] Huang P Liu T Huang L Liu H Lei M Xu W. Use of chest CT in combination with negative RT-PCR assay for the 2019 novel coronavirus but high clinical suspicion. Radiology. (2020) 295:22–3. 10.1148/radiol.2020200330 32049600 PMC7233360

[99] Ai T Yang Z Hou H Zhan C Chen C Lv W. Correlation of chest CT and RT-PCR testing in coronavirus disease 2019 (COVID-19) in China: a report of 1014 cases. Radiology. (2020). 10.1148/radiol.2020200642. [Epub ahead of print]. 32101510 PMC7233399

[100] Feng X Tao X Zeng L Wang W Li G. [Application of pulmonary ultrasound in the diagnosis of COVID-19 pneumonia in neonates]. Chin J Pediatr. (2020) 58. 10.3760/cma.j.cn112140-20200228-00154 32392948

[101] Freije CA Myhrvold C Boehm CK Lin AE Welch NL Carter A. Programmable inhibition and detection of RNA viruses using Cas13. Mol Cell. (2019) 76:826–37.e11. 10.1016/j.molcel.2019.09.013 31607545 PMC7422627

[102] Nguyen TM Zhang Y Pandolfi PP. Virus against virus: a potential treatment for 2019-nCov (SARS-CoV-2) and other RNA viruses. Cell Res. (2020) 30:189–90. 10.1038/s41422-020-0290-0 32071427 PMC7054296

[103] Favre G Pomar L Qi X Nielsen-Saines K Musso D Baud D. Guidelines for pregnant women with suspected SARS-CoV-2 infection. Lancet Infect Dis. (2020). 10.1016/S1473-3099(20)30157-2. [Epub ahead of print]. 32142639 PMC7134390

[104] Medical Association of Chinese People's Liberation Army; Editorial Committee of Chinese Journal of Contemporary Pediatrics; Preparatory Group of Pediatric Disaster Pediatric Society Chinese Medical Association [Response plans in the neonatal intensive care unit during epidemic of SARS-CoV-2 infection (2nd Edition)]. Zhongguo Dang Dai Er Ke Za Zhi. (2020) 22:205–10. 10.7499/j.issn.1008-8830.2020.03.005 32204754 PMC7389594

[105] Ong SWX Tan YK Chia PY Lee TH Ng OT Wong MSY Air, surface environmental, and personal protective equipment contamination by severe acute respiratory syndrome coronavirus 2 (SARS-CoV-2) from a symptomatic patient. JAMA. (2020) 323:1610–2. 10.1001/jama.2020.3227 PMC7057172 32129805

[106] Xie X Xue Q Zhou Y Zhu K Liu Q Zhang J. Mental health status among children in home confinement during the coronavirus disease 2019 outbreak in Hubei Province, China. JAMA Pediatr. (2020). 10.1001/jamapediatrics.2020.1619. [Epub ahead of print]. 32329784 PMC7182958

[107] He Y Wang Z Li F Shi Y. Public health might be endangered by possible prolonged discharge of SARS-CoV-2 in stool. J Infect. (2020) 80:e18–9. 10.1016/j.jinf.2020.02.031 32145217 PMC7133677

[108] Marraro GA Spada C. Consideration of the respiratory support strategy of severe acute respiratory failure caused by SARS-CoV-2 infection in children. Zhongguo Dang Dai Er Ke Za Zhi. (2020) 22:183–94. 10.7499/j.issn.1008-8830.2020.03.002 32204751 PMC7389599

[109] Chen T Wu D Chen H Yan W Yang D Chen G. Clinical characteristics of 113 deceased patients with coronavirus disease 2019: retrospective study. BMJ. (2020) 368:m1091. 10.1136/bmj.m1295 32217556 PMC7190011

[110] Holshue ML deBolt C Lindquist S Lofy KH Wiesman J Bruce H. First case of 2019 novel coronavirus in the United States. N Engl J Med. (2020) 382:929–36. 10.1056/NEJMoa2001191 32004427 PMC7092802

[111] de Wit E Feldmann F Cronin J Jordan R Okumura A Thomas C. Prophylactic and therapeutic remdesivir (GS-5734) treatment in the rhesus macaque model of MERS-CoV infection. Proc Natl Acad Sci USA. (2020) 117:201922083. 10.1073/pnas.1922083117 32054787 PMC7104368

[112] Cao B Wang Y Wen D Liu W Wang J Fan G. A trial of lopinavir-ritonavir in adults hospitalized with severe covid-19. N Engl J Med. (2020) 382:1787–99. 10.1056/NEJMoa2001282 32187464 PMC7121492

[113] WHO Clinical management of severe acute respiratory infection when COVID-19 is suspected. Available online at: https://www.who.int/publications-detail/clinical-management-of-severe-acute-respiratory-infection-when-novel-coronavirus-(ncov)-infection-is-suspected (accessed Mar 13, 2020).

[114] Russell CD Millar JE Baillie JK Clinical evidence does not support corticosteroid treatment for 2019-nCoV lung injury. Lancet. (2020) 395:473–5. 10.1016/S0140-6736(20)30317-2 32043983 PMC7134694

[115] Shang L Zhao J Hu Y Du R Cao B. On the use of corticosteroids for 2019-nCoV pneumonia. Lancet. (2020) 395:683–4. 10.1016/S0140-6736(20)30361-5 32122468 PMC7159292

[116] Zhao JP Hu Y Du RH Chen ZS Jin Y Zhou M [Expert consensus on the use of corticosteroid in patients with 2019-nCoV pneumonia]. Zhonghua Jie He He Hu Xi Za Zhi. (2020) 43:E007 10.3760/cma.j.issn.1001-0939.2020.0007 32164084

[117] The Society of Pediatrics Chinese Medical Association; the Editorial Board Chinese Journal of Pediatrics [Recommendations for the diagnosis, prevention and control of the 2019 novel coronavirus infection in children (first interim edition)]. Chin J Pediatr. (2020) 58:169–74. 10.3760/cma.j.issn.0578-1310.2020.0004 32135584

[118] Wang Z Chen X Lu Y Chen F Zhang W Clinical characteristics and therapeutic procedure for four cases with 2019 novel coronavirus pneumonia receiving combined Chinese and Western medicine treatment. Biosci Trends. (2020) 4:64–8. 10.5582/bst.2020.01030 32037389

[119] Walls AC Park YJ Tortorici MA Wall A McGuire AT Veesler D. Structure, function and antigenicity of the SARS-CoV-2 spike glycoprotein. Cell. (2020) 181:281–92.e6. 10.1101/2020.02.19.956581 32155444 PMC7102599

[120] Hoffmann M Kleine-Weber H Schroeder S Müller MA Drosten C Pohlmann S. SARS-CoV-2 cell entry depends on ACE2 and TMPRSS2 and is blocked by a clinically proven protease inhibitor. Cell. (2020) 181:271–80.e8. 10.1016/j.cell.2020.02.052 32142651 PMC7102627

[121] Chen L Xiong J Bao L Shi Y. Convalescent plasma as a potential therapy for COVID-19. Lancet Infect Dis. (2020) 20:398–400. 10.1016/S1473-3099(20)30141-9 32113510 PMC7128218

[122] Shen C Wang Z Zhao F Krüger N Herrler T Erichsen S Treatment of 5 critically Ill patients with COVID-19 with convalescent plasma. JAMA. (2020) 323:1582–9. 10.1001/jama.2020.4783 PMC7101507 32219428

[123] Yang X Yu Y Xu J Shu H Xia J Liu H. Clinical course and outcomes of critically ill patients with SARS-CoV-2 pneumonia in Wuhan, China: a single-centered, retrospective, observational study. Lancet Respir Med. (2020) 8:471–81. 10.1016/S2213-2600(20)30079-5 32105632 PMC7102538

[124] Li AM Ng PC. Severe acute respiratory syndrome (SARS) in neonates and children. Arch Dis Child Fetal Neonatal Ed. (2005) 90:F461–5. 10.1136/adc.2005.075309 16244207 PMC1721969

[125] McCloskey B Heymann DL. SARS to novel coronavirus - old lessons and new lessons. Epidemiol Infect. (2020) 148:e22. 10.1017/S0950268820000254 32019614 PMC7026896

[126] Yang ZD Zhou GJ Jin RM Zhi-Sheng L Zong-Qi D Xiong X Clinical and transmission dynamics characteristics of 406 children with coronavirus disease 2019 in China: a review. J Infect. (2020). 10.1016/j.jinf.2020.04.030. [Epub ahead of print]. PMC7187854 32360500

